# Pronounced Trace Element Variation in Follicular Fluids of Subfertile Women Undergoing Assisted Reproduction

**DOI:** 10.3390/nu13114134

**Published:** 2021-11-19

**Authors:** Lilly Johanna Schmalbrock, Gregor Weiss, Eddy Rijntjes, Nina Reinschissler, Qian Sun, Michael Schenk, Lutz Schomburg

**Affiliations:** 1 Cardiovascular–Metabolic–Renal (CMR)—Research Center, Institut für Experimentelle Endokrinologie, Charité–Universitätsmedizin Berlin, Corporate Member of Freie Universität Berlin and Humboldt Universität zu Berlin, Hessische Straße 3-4, D-10115 Berlin, Germany; lilly.schmalbrock@charite.de (L.J.S.); eddy.rijntjes@charite.de (E.R.); qian.sun@charite.de (Q.S.); 2 Das Kinderwunsch Institut Schenk GmbH, Am Sendergrund 11, A-8143 Dobl, Austria; gregor.weiss@kinderwunsch-institut.at (G.W.); nina.reinschissler@gmx.at (N.R.)

**Keywords:** infertility, PCOS, trace elements, selenium

## Abstract

Female subfertility is a growing concern, especially in view of an increasing prevalence of polycystic ovary syndrome (PCOS). Assisted reproductive technologies (ART) offer a perspective for pregnancy, but the outcome rate is still suboptimal. The trace elements (TE), copper (Cu), selenium (Se), and zinc (Zn) are essential for fertility and development. We hypothesized that TE concentrations are related to oocyte quality and growth and affect pregnancy outcomes in women undergoing ART. Concentrations of TE were measured by total reflection X-ray fluorescence. Extracellular glutathione peroxidase 3 (GPX3) and selenoprotein P (SELENOP) were determined as additional Se biomarkers. Corresponding serum and follicular fluid (FF) samples were available from women with (*n* = 20) and without (*n* = 20) PCOS diagnosis undergoing hormone treatment within the ART procedure, respectively, and FF samples were classified into five groups based on morphological assessment. Serum showed higher TE concentrations than FF, and TE levels correlated positively between both matrices. Individual FF from the same women showed surprisingly high variability in TE concentration, and follicles without oocytes displayed the lowest TE concentrations. Both Se biomarkers GPX3 and SELENOP were present in FF and correlated positively to Se concentrations. Some notable relationships were observed between morphokinetic parameters, TE concentrations, and GPX3 activity. A slightly depressed serum Zn concentration was observed in PCOS. Our results indicate a direct relationship between TE in serum and FF, positive correlations between the three Se biomarkers in FF, and high variability between the FF from the same woman with the lowest TE concentrations in the follicles with the poorest quality. The differences observed in relation to PCOS diagnoses appear relatively minor. Collectively, the data support the notion that TE assessment of follicles may contribute to optimal oocyte selection and subsequently influence ART success.

## 1. Introduction

An estimated 48 to 72 million couples worldwide suffer from infertility, around half of which lack an explanation for their failure in conceiving. Among the reasons leading to female subfertility are primary and secondary ovulatory dysfunction, such as ovarian insufficiency or polycystic ovary syndrome (PCOS) [1,2]. PCOS is one of the most common hormonal disorders of women of reproductive age, negatively affecting fertility in about 75% of patients [3]. PCOS is associated with low-grade inflammation and metabolic comorbidities [4]. There are indications that lifestyle modifications with weight loss prior to assisted reproductive technologies (ART) show benefits compared to immediate treatment in obese women with PCOS [5]. Yet, ART is a promising option with suitable interventions for the clinical management of infertility, including in vitro-fertilization (IVF) and intracytoplasmic sperm injection (ICSI) [6]. With a steadily rising demand over the past decades, it is essential to understand which factors may optimize the outcome of ART treatment, as live birth rate (LBR) is still observed in around 36% to 50% of attempts only [7,8]. One challenging step during ART is the selection of the optimal embryo for transfer. This is traditionally performed using microscopic evaluation of morphological parameters at predefined time points (“Gardner Schoolcraft criteria”) [9]. The inclusion of additional parameters determined by modern time-lapse imaging (TLI) of the blastomere and embryo developmental kinetics (“morphokinetics”) are increasingly used during quality assessment of preimplantation embryos [9,10]. As in the case of conventional assessment, TLI is also somewhat limited in selecting the optimal embryo for a high chance of successful pregnancy and live birth [11]. The follicular fluid (FF) surrounding the oocyte creates a special microenvironment for oocyte maturation and development [12]. Alterations in the microenvironment of oocytes in the FF have been suggested to influence oocyte quality from maturation to fertilization, early embryo development, and subsequent pregnancy course [13]. Experimental and clinical studies indicate that growth and fertility are strongly affected by trace element (TE) supply, and deficiencies are known to impair both male and female gametogenesis as well as embryonic development [14,15,16,17,18]. In particular, the trace elements copper (Cu), selenium (Se), and zinc (Zn) have emerged as the most essential micronutrients for both female and male fertility [19,20,21,22,23].

The effects of TE deficiencies on spermatogenesis and sperm characteristics have been studied in large detail. The selenoenzyme glutathione peroxidase 4 (GPX4) is synthesized in spermatids and becomes transformed into a structural protein of the mitochondrial sheath of spermatozoa [24]. The supply of Se to testes is mediated by the Se transport protein selenoprotein P (SELENOP). Genetic inactivation of *Selenop* causes male infertility, and recombinant expression of a *SELENOP* transgene in the liver restores Se supply and male fertility [25]. A specific receptor in testes mediates the directed and efficient uptake of SELENOP and thereby maintains Se supply to growing sperm, namely the apolipoprotein E receptor-2 (ApoER2) [26]. In addition, high concentrations of SELENOP have been detected in seminal fluid, likely for protecting sperm on their journey toward fertilization [27]. Collectively, a tightly regulated and complex system has been developed in the male to enable and maintain Se supply for fertility, involving SELENOP as a specific transport molecule and APOER2 as an efficient receptor for Se uptake. In comparison, our understanding of the molecular details of Se supply to follicles is limited, and the interaction of the essential micronutrients with oocyte maturation is poorly understood, especially in relation to PCOS. Improved knowledge and a better database may be of immediate relevance for ART and support the attempts to improve the selection of the optimal oocyte for fertilization. In order to close some aspects of the research gap, we determined Cu, Se, and Zn along with additional informative protein biomarkers of Se status in FF of women with or without a diagnosis of PCOS undergoing ART and compared the results to corresponding serum concentrations to identify potential interactions. We hypothesized that the TE status in serum and FF may correlate to the quality characteristics of an oocyte.

## 2. Materials and Methods

### 2.1. Study Population

Serum and FF samples from women undergoing ART at the Kinderwunsch Institut Schenk GmbH in Dobl, Austria, were collected, stored, and retrieved from the Biobank Graz of the Medical University of Graz, Austria. All women agreed to sampling, storage, and later retrospective scientific analysis, and written informed consent was obtained from each woman prior to sampling. Sample collection and research were approved by the ethical committee of the Medical University of Graz (approval # 20–492 ex08/09). The collection of biosamples was established under the highest quality standards and transparency [28]. Serum samples from twenty women with a diagnosis of PCOS (PCOS group) and from a further twenty women without PCOS (subfertile group) along with up to ten FF per patient were included in the analyses. PCOS was diagnosed according to the Rotterdam criteria as described [29,30], and a set of relevant clinical characteristics were determined (Table 1). At the time of sampling, 8 women in the subfertile and 15 in the PCOS group were diagnosed as primary infertile. A prior pregnancy or a known and diagnosed infertility of the male partner were not applied as an exclusion criterion. Women in the PCOS group were, on average, slightly younger than those in the subfertility group, BMI was similar, and only a small number of women were active smokers (Table 1). Serum anti-Mullerian hormone (AMH) concentrations were higher in the PCOS group than in the subfertile group, whereas estradiol (E2) concentrations were similar. Both hormones were determined using fully automated electrochemiluminescence immunoassays (ECLIA) for quantitative determination (Cobas-e411, Roche Diagnostics GmbH, Penzberg, Germany).

### 2.2. Sample Preparation and Characterization of Oocytes

**Figure 1:**
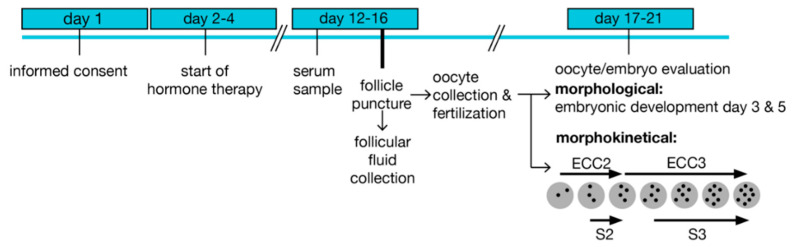
Treatment scheme and sample preparation. Enrolment started on day 1 before the initiation of hormone treatment. A serum sample was prepared prior to follicular puncture and follicular fluid collection. Oocytes and embryos were evaluated based on morphological characteristics, and embryos were cultured in time-lapse systems after fertilization for recording morphokinetic times. ECC; embryo cell cycle, s; synchronization within an ECC.

The samples were obtained following a standardized protocol (Figure 1). Controlled ovarian hyperstimulation was initiated by the administration of follicle-stimulating hormone (FSH) at days 2–4 of the menstrual cycle, and a GnRH antagonist was applied on days 5–6. Ovulation was induced by chorion gonadotropin (hCG) injection 35 h before transvaginal puncture of mature follicles (diameter >15 mm) under ultrasound control at day 12–16. Ten FF were collected per woman and preserved individually, as described [28]. Briefly, follicles larger than 10 mm in diameter were aspirated under transvaginal ultrasound guidance (GE Healthcare Austria GmbH, Pfaffing, Austria) with a Steiner-Tan needle 17 gauge and a Steiner flush/valve (IVFETFLEX.com HandelsgmbH & Co KG, Graz, Austria). Follicular fluid was examined for oocytes in an IVF workstation L24E with the heating stage (K-SYSTEMS Kivex Biotec A/S, Birkerød, Denmark) under constant conditions of 37 °C and subsequently individually stored at −80 °C. Fertilization was performed 1–4 h after oocyte retrieval. Embryos were cultured in time-lapse systems and transferred (or cryopreserved) on day 3 or 5 after fertilization. A pregnancy test was carried out 10 days after embryo transfer (ET).

**Figure 2:**
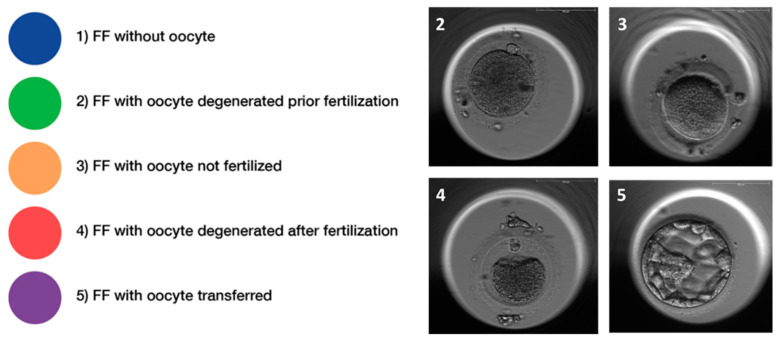
Classification of follicular fluid samples. Follicular fluids (FF) were retrospectively classified according to morphological characteristics of the oocyte and/or embryo and classified into five distinctive quality classes, i.e., (1) FF without an oocyte, (2) FF with an oocyte, which degenerated prior to fertilization, (3) FF with an oocyte that could not be fertilized, (4) FF with an oocyte, which degenerated after fertilization, and (5) FF with a successfully fertilized oocyte and transferred or frozen embryo. Representative pictures of oocytes (class 2–4) and a blastocyst (class 5), respectively, are provided in the panel to the left.

Embryo quality was evaluated at development day 3 with regards to the symmetry of the cells and the percentage of fragmentation. The expansion of the blastocyst, the quality of the inner cell mass (ICM), and the quality of the trophectoderm were determined on embryonic day 5 as described [31]. According to these characteristics, the corresponding FF were divided into five groups (Figure 2). Seventy follicular fluid samples were excluded as no clear allocation to one group was possible due to the presence of more than one oocyte in the corresponding follicle or due to missing information (oocyte sharing), including all the FF samples from two women.

Complementary to morphological variables, morphokinetic parameters were collected by time-lapse technology (embryoscope), as described [32]. The duration of embryo cell cycles (ECC), i.e., doubling of the cell number of blastomeres, was recorded and annotated using the proposed guidelines and terminology [33]. ECC2 describes the duration of the second cell cycle, which results in the doubling from two to four cells (t4–t2), and ECC3 describes the third cell cycle (t8–t4), t_n_ describing the time point of the first observation of the respective numbers (*n*) of discrete cells. Synchronization (s) describes the time between the next geometric sequence within an ECC, with s2 as the synchronicity time of the sisters’ blastomere division within EEC2, calculated as t4–t3. Analogously for s3, the synchronicity of the four blastomere divisions within ECC3, calculated as t8–t5 [33].

### 2.3. Trace Element (TE) Analysis in Serum and FF Samples

The laboratory analyses of TE status were conducted at a remote site from the biobank (Institute for Experimental Endocrinology, Charité-Universitätsmedizin Berlin, Germany) by scientists blinded to the clinical data. Concentrations of TE in serum and FF were determined by total reflection X-ray fluorescence (TXRF) analysis using a benchtop TXRF analyzer (S4 T-STAR, Bruker nano GmbH, Berlin, Germany), as described [34,35]. Briefly, serum or FF samples were diluted with an equal volume of a gallium standard (1000 µg Ga/L), mixed thoroughly, and applied to polished quartz glass disks. Samples were dried overnight, and a standard sample from a pool of 25 healthy donor sera served as a control in each analysis run. The inter-assay coefficient of variation was below 10%.

Enzymatic activity of GPX3 was determined in serum and FF samples by a coupled optic test procedure monitoring glutathione reductase-catalyzed consumption of nicotinamide adenine dinucleotide phosphate (NADPH) at 340 nm and 25 °C [36,37]. Briefly, samples of 5 µL were applied to 96-well plates, and a reaction mixture (200 µL) of 1 mM NaN_3_, 3.4 mM reduced glutathione, 0.3 U/mL glutathione reductase, and 0.27 mg/mL NADPH was added. The test was started with 10 µL of hydrogen peroxide (0.00375%). The decrease in NADPH absorbance at 340 nm is proportional to GPX3 activity. A constant serum sample was included in each assay run for quality control and yielded an inter- and intra-assay CV below 20%.

The concentration of SELENOP was determined by a commercially available sandwich ELISA (selenOtest, selenOmed GmbH, Berlin, Germany) as described earlier [38]. SDS-PAGE was performed with serum and FF samples using 12.5% Bis-Tris gels in combination with a Tris/SDS running buffer as described [38]. The proteins were blotted onto a 0.2 µm PVDF membrane (Thermo Fisher Scientific) in transfer buffer (250 mM Tris, 1.92 M glycine, 1% SDS, pH 8.3) containing 20% methanol. PonceauS (Sigma-Aldrich Biochemie GmbH, Hamburg, Germany) was used for protein visualization after transfer. SELENOP was detected by Western blot using anti-human SELENOP antibodies (selenOmed GmbH, Berlin, Germany) and an anti-mouse IgG-HRP from sheep (Amersham BioSciences UK Ltd., Amersham Pl, Little Chalfont Buckinghamshire, U.K.) in combination with the ECL Western Blotting Detection Reagents and Hyperfilm™ (GE Healthcare GmbH, Solingen, Germany). Quantification of relative SELENOP expression was achieved by using the image-processing program ImageJ (NIH, Bethesda, MD, USA, version 1.53i).

### 2.4. Statistical Analysis

Statistical analyses were performed using the Statistical Package for the Social Sciences (IBM SPSS Statistics 25, Chicago, IL, USA) and GraphPad Prism 7 (GraphPad Software Inc., San Diego, CA, USA). Parametric tests were used since TE concentrations are usually normally distributed. Interrelations between parameters were analyzed by Pearson correlation test. Differences in morphokinetic times were tested using paired *t*-test. Differences between the PCOS and subfertile groups were tested using an unpaired *t*-test or a linear mixed model test. The linear mixed models included age as covariate and group (PCOS/subfertile) and follicular quality (when studying FF) as fixed effects of testing the interaction. The subjects were included as random effects. Bonferonni post-hoc tests were used to correct for multiple testing.

## 3. Results

### 3.1. Patient Characteristics

A total of *n* = 20 women with POCS (PCOS) and *n* = 20 women with other reasons for reduced fertility (subfertile) were included in this observational study. Women in the PCOS group were on average significantly younger as compared to the subfertile group. Eight women of the subfertile and seven women of the PCOS group achieved pregnancy, and seven in the subfertile versus five in the PCOS group achieved a successful live birth (Table 1), which resembles a pregnancy rate of 40% and 35%, respectively.

### 3.2. Trace Element Status in Serum and Follicular Fluids (FF)

**Figure 3:**
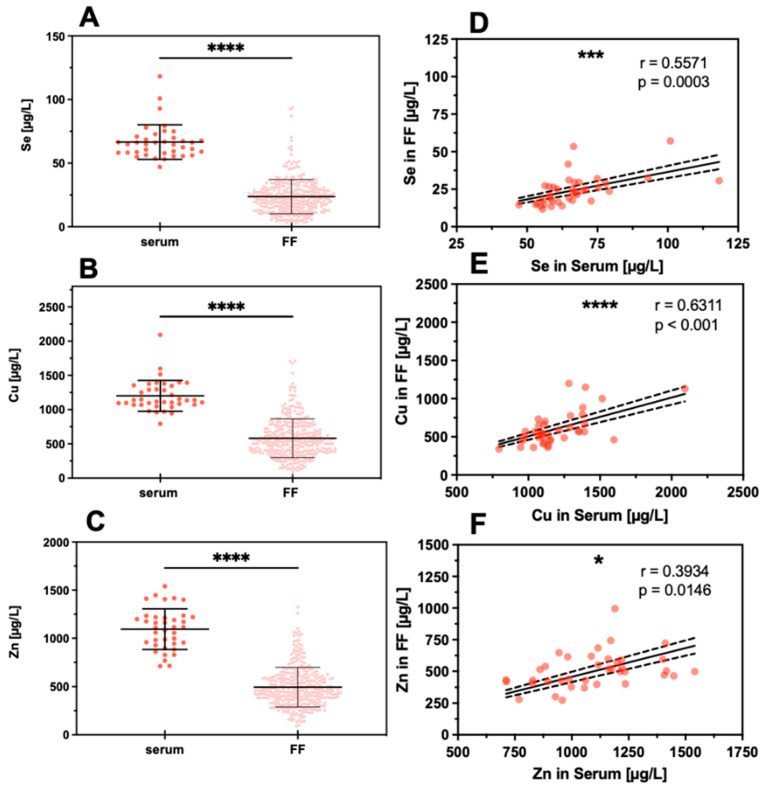
Comparison of TE concentrations in serum and FF samples. Trace elements were determined in serum samples (serum, *n* = 38) and follicular fluids (FF, *n* = 324 for Se, *n* = 330 for Cu, *n* = 330 for Zn). On average, the concentrations of (**A**) Se, (**B**) Cu, and (**C**) Zn were significantly lower in FF as compared to serum. Despite this difference, a significant linear positive correlation was observed between the FF and serum samples for (**D**) Se, (**E**) Cu, and (**F**) Zn. Samples were compared by paired *t*-test, and the significance shown for (**A**–**C**) was calculated based on the means of TE concentrations in the FF from the same woman; significance is indicated by * *p* < 0.05, *** *p* < 0.001, and **** *p* < 0.0001.

The concentration of Cu, Se, and Zn were determined in the serum and FF samples. The direct comparison indicates that all three TE are on average considerably lower in the FF than in the serum samples (Figure 3A–C). Evaluating the interrelation between TE concentrations in the matrices, i.e., serum and FF, strong positive correlations for Se and Cu and a less stringent correlation for Zn were detected (Figure 3D–F).

### 3.3. Strong Variability of Trace Element Status in Follicular Fluids versus Corresponding Serum

**Figure 4:**
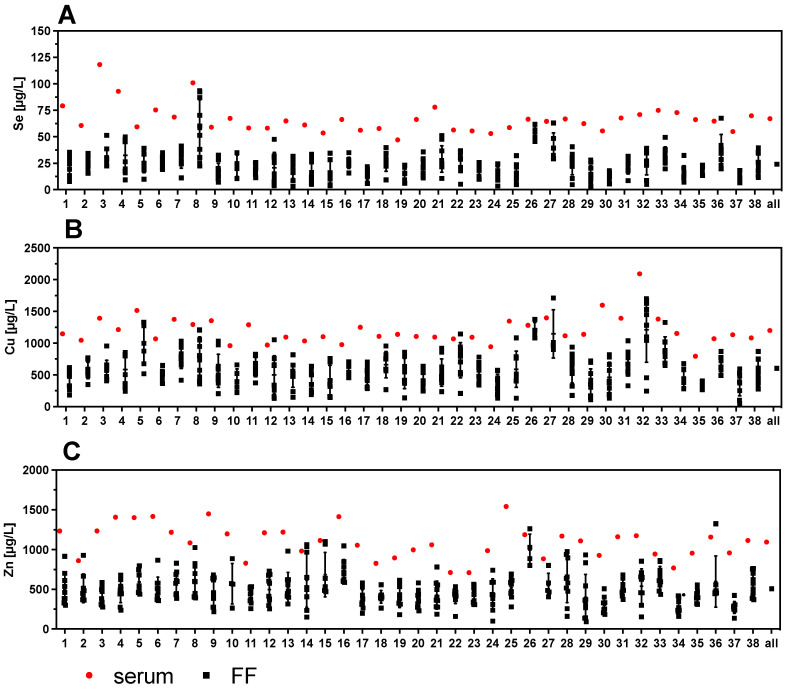
Comparison of TE concentrations in serum and corresponding follicular fluids (FF). Trace element concentrations of one serum sample with up to 10 corresponding FF were compared per patient. The results indicate a wide variation in TE concentrations in the different FF with regards to (**A**) Se, (**B**) Cu, and (**C**) Zn. Samples 20–37 correspond to women with PCOS.

One serum sample and up to ten corresponding FF per woman were available for analysis. The direct comparison of the TE indicates a wide variety of concentrations in the different FF obtained from the same woman (Figure 4). The variation in TE concentrations of the FF within one woman can be higher than the variation between all the serum samples analyzed across the women. Notably, some FF are almost reaching serum concentrations (e.g., samples #8, #12, #26, #27, or #36 in case of Se), while other FF sample sets from a particular woman show a more uniform and relatively low trace element status (e.g., samples #2, #6, #11, #35, or #37 in case of Se) (Figure 4A). The same pronounced variation of concentrations and differences also applies to Cu and Zn (Figure 4B,C).

**Figure 5:**
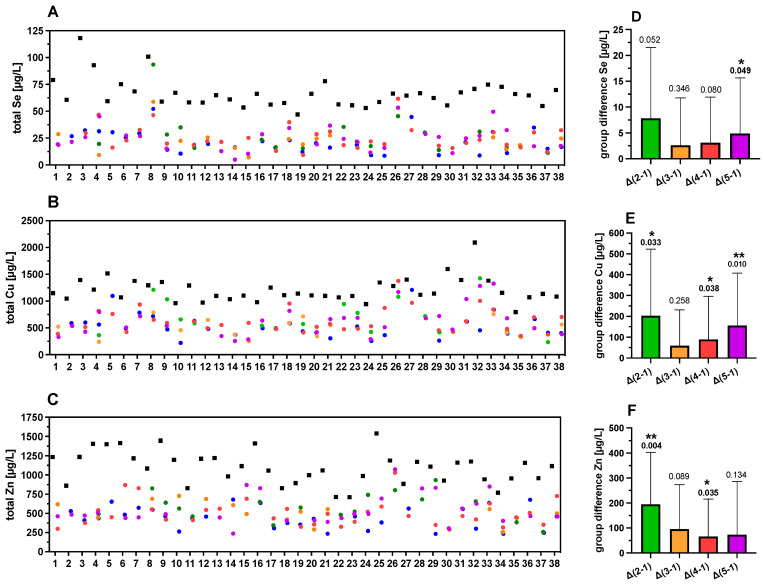
TE concentrations in FF in relation to quality characteristics. The FF are color-coded according to quality characteristics; group 1 (blue, poor quality), group 2 (green), group 3 (orange), group 4 (red), and group 5 (purple, highest quality), as described above (Figure 2). Strong differences between corresponding serum and FF samples and between the different FF of varying quality from the same woman are observed for (**A**) Se, (**B**) Cu, and (**C**) Zn. The mean differences in TE concentrations of the groups of FF with different quality (group differences) with respect to group 1 as reference (poor quality) are indicated for (**D**) Se, (**E**) Cu, and (**F**) Zn. In general, higher TE concentrations are observed in serum than in FF and among the FF with increasing quality. Samples 20–37 correspond to women with PCOS. Statistical significance is indicated by * *p* < 0.05, and ** *p* < 0.01.

To analyze the strong variability of TE concentrations in the FF further and to relate it to follicle quality, the samples were divided into five groups of different characteristics, based on morphological quality as described above (Figure 2 and Figure 5A–C). The TE concentrations in group 1 (FF without an oocyte) were used as reference. Mean differences (MD) in TE concentrations between follicles in group 2–5 with group 1 were calculated and denoted as Δ groups (Δ2–1, Δ3–1, Δ4–1 or Δ5–1) (Figure 5D–F). The Se concentrations showed a significant difference when comparing the FF of the highest quality (group 5) with group 1 of follicles without an oocyte, denoted as “Δ5–1” (Figure 5D). For Cu, three quality groups showed higher concentrations as compared to group 1 (Figure 5E), whereas Zn concentrations were significantly higher in groups 2 and 4 as compared to group 1 (Figure 5F). Collectively, the data indicate that the FF with poor quality (group 1) showed generally lower TE concentrations than the FF with better oocytes in groups 2–5. However, the trends of higher TE concentrations with FF quality were not uniformly observed across all sample sets.

### 3.4. Protein Biomarkers of Se Status in Follicular Fluids and Corresponding Serum Samples

**Figure 6:**
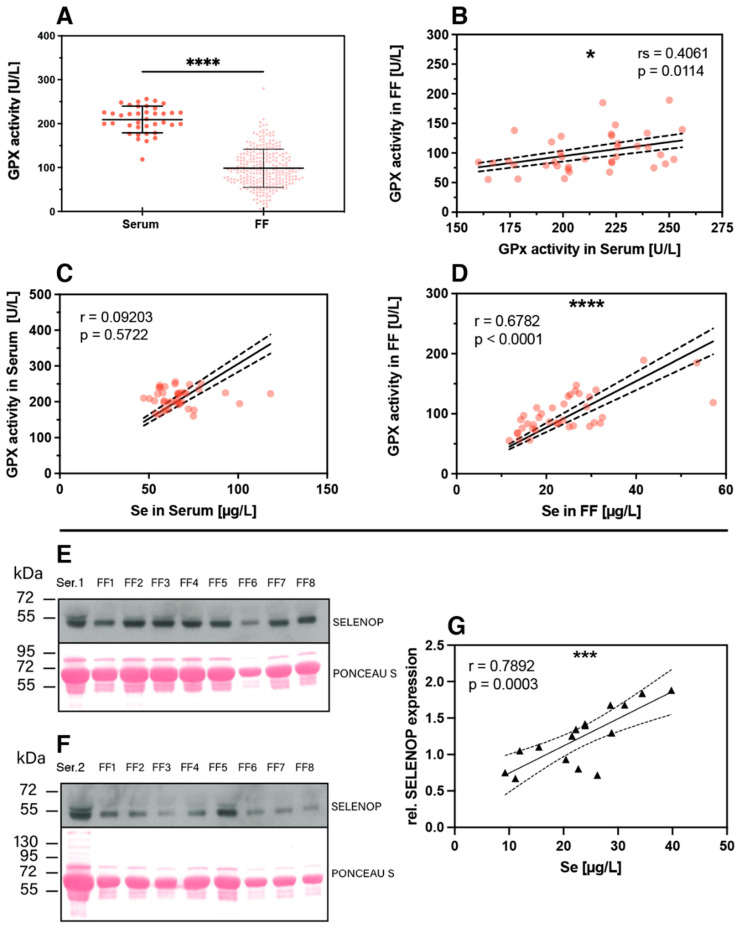
Protein biomarkers of Se status in FF and serum. (**A**) Expression of the extracellular glutathione peroxidase (GPX3) was analyzed in the serum and FF samples and showed lower and very variable enzymatic activity in FF. Analysis of significance was conducted with the mean FF GPX3 activity from the same woman with the corresponding serum by paired *t*-test. (**B**) The GPX3 activities in serum and corresponding FF samples from the same woman showed a linear and positive correlation. (**C**) The correlation between serum GPX3 activity and total serum Se was poor, whereas (**D**) GPX3 activity and total Se concentration in the FF correlated strongly and linearly. (**E**,**F**) Western blot analysis indicates SELENOP expression in the FF samples at the same size and band pattern at 50–55 kDa as in the corresponding serum sample; analysis was conducted in the sample sets of one subject selected from the group of subfertile (**E**) and PCOS (**F**) women each. Ponceau S staining illustrates the loading of the lanes for improved comparison of signal strengths. (**G**) A positive linear correlation is observed for SELENOP and total Se in the FF samples. Statistical significance is indicated by * *p* < 0.05, *** *p* < 0.001 and **** *p* < 0.0001.

The nutritional supply with the trace element Se can be assessed by different selenoproteins as additional biomarkers of Se status [39,40], and a combination of these biomarkers may provide particularly meaningful prognostic information [41]. In this study, two complementary biomarkers were determined in addition to total Se in the serum and FF samples, i.e., extracellular glutathione peroxidase (GPX3) enzymatic activity and the concentration of the extracellular Se transporter selenoprotein P (SELENOP). In line with the findings of reduced total Se in FF compared to serum, also significantly lower GPX3-activity was observed in FF as compared to serum (Figure 6A). Notably, a linear positive correlation was observed for GPX3 activity in serum and in the corresponding FF samples from the same woman (Figure 6B). The GPX3 activity in serum of the different women was relatively constant and poorly related to serum Se (Figure 6C), whereas a strong and linear correlation was observed between GPX3 activity and Se concentrations in FF (Figure 6D). This finding raises the question of whether Se in FF is controlled and dominated by GPX3, with no or only minor contribution from the Se transporter SELENOP. However, Western blot analysis detected immunoreactive SELENOP at the expected size of 50–55 kDa in FF, and the quantitative analysis showed a strong positive linear correlation between SELENOP concentration in FF and total FF Se concentrations (Figure 6E–G).

### 3.5. Trace Element Status in Patients in Relation to a Diagnosis of PCOS

**Figure 7:**
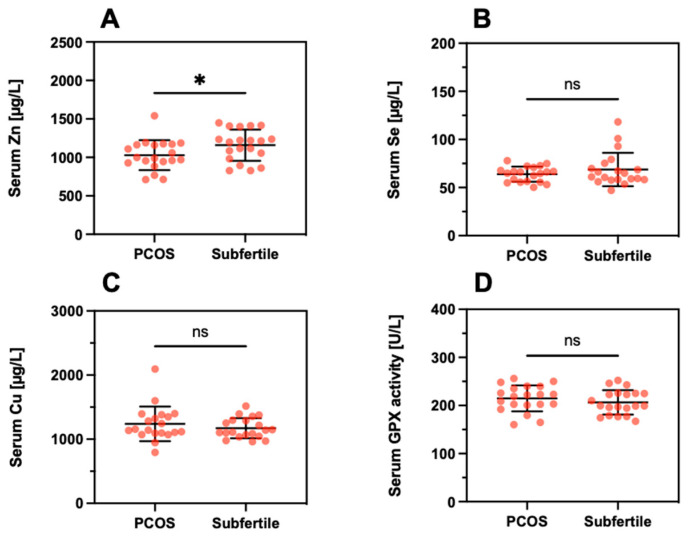
Serum trace element concentrations and GPX3 activity in relation to PCOS. The direct comparison of serum (**A**) Zn, (**B**) Se, (**C**) Cu, and (**D**) GPX3 activity indicates minor differences only, with the notable exception of a slightly suppressed serum Zn concentration in PCOS. Tested with a linear mixed model; * indicates *p* < 0.05. ns indicates no significant difference.

Next, differences in TE concentrations were analyzed between the subfertile women with respect to a diagnosis of PCOS. A direct comparison of the serum samples indicated a slightly lower Zn concentration in PCOS (PCOS; 1029 ± 195 µg/L vs. Subfertile; 1159 ± 203, *p* = 0.046, Figure 7A). No significant differences were observed for serum Se (Figure 7B), serum Cu (Figure 7C), or serum GPX3 activity (Figure 7D).

Regarding the TE concentrations in relation to quality characteristics of the FF, the data were compared per woman according to the differences in relation to group 1 (poor quality) serving as a reference. Conducting these analyses, few significant differences were noted (Table 2). The FF samples from the group of women with PCOS showed slightly lower Zn concentrations in group 3 (PCOS; 419 ± 142 vs. Subfertile; 571 ± 111; *p* = 0.029) and elevated Cu concentrations in group 5 (PCOS 742 ± 318 vs. Subfertile; 507 ± 190; *p* = 0.039). No other significant differences were observed. The differences in trace element concentrations and GPx activity were tested using a linear mixed model. Delta groups, based on the means per woman, were tested using unpaired *t*-tests.

A slightly lower Zn and an elevated Cu concentration were detected in groups 3 and 5, respectively, of samples from the women with a PCOS diagnosis in comparison to the other group of subfertile women. With regards to differences between the quality characteristics of FF, significantly elevated Cu and Zn concentrations were observed in the high-quality group (group 5) compared to TE concentration in FF without an oocyte (group 1) in the samples from women with PCOS as compared to the samples of subfertile women (Table 2). This result indicates that the observed differences between group 5 and group 1 for the Zn and Cu concentrations mainly refer to PCOS women or are at least more pronounced in PCOS than in FF from women with other reasons for subfertility.

### 3.6. Trace Element Status in Relation to Morphokinetic Variables

Besides the morphological parameters, morphokinetic variables of the oocytes were measured, including the timings of cleavage and specific early developmental endpoints among embryos. Timing of embryo cell cycles two and three (ECC2;ECC3) and times of synchronization (s2,s3) within the ECCs were calculated for groups 4 and 5. The time to reach synchrony after initiation of the second and third cleavage (s2 and s3) was significantly shorter in group 5 vs. group 4 (s2, group 4; 5.2 ± 5.8 vs. group 5; 1.8 ± 2.6 h, *p* = 0.023, and s3, group 4; 15.7 ± 9.1 vs. group 5; 8.7 ± 4.8 h, *p* = 0.017). Times of ECC2 and ECC3 were also shorter in group 5 compared to group 4, but without reaching statistical significance (ECC2, group 4; 13.6 ± 6.0 vs. group 5; 12.6 ± 1.5 h; ECC3, group 4; 22.7 ± 7.3 vs. group 5; 19.1 ± 3.5 h) (Table 3).

When correlating TE concentrations with the calculated times, some notable positive and negative correlations for Cu, Zn, Se, and GPx3 activity were observed (Supplementary Table S1). In group 4, Cu and Se correlated with s3 (Cu; *n* = 15, r = 0.443, *p* = 0.03, and Se; *n* = 15, r = 0.476, *p* = 0.016). In group 5, Zn and Se correlated with ECC2 (*n* = 19, r = 0.402, *p* = 0.042, and Se; *n* = 19, r = 0.39, *p* = 0.049). A stringent correlation of GPX3-activity in group 4 with s3 was detected (*n* = 15 r = 0.648, *p* = 0.001), in line to the correlation of Se in group 4 with s3. In these analyses of TE concentrations with morphokinetic times, no significant differences were found between the women in the PCOS or subfertile group.

## 4. Discussion

This study reports biomarkers of TE status in matched samples of serum and FF from women undergoing ART, with a focus on the trace element Se. In general, positive correlations between serum and FF were observed, and a tendency of relatively low TE concentrations was noted in FF of the lowest quality (group 1). In view of our standardized and well-controlled sampling [28], the data highlight a hitherto unexpected high variation in TE status between the different follicles of the same woman, verified in the case of Se by three complementary biomarkers including two Se-dependent proteins. The molecular reasons for this diversity in TE biomarkers are unknown but support the notion that the FF composition is controlled by follicle cells and the extent of exchange with the circulation and degree of plasma transudate. In view of the positive correlation of TE biomarkers in serum and FF, deficiencies in intake seem to be translated directly into the FF, and an improved TE supply would thus not only raise serum TE concentrations but likely also the TE status in the FF as a promising adjuvant measure during ART. In contrast to our expectations, there were no strong differences in serum and FF when separating the patients according to PCOS, but the decreased Zn concentration in serum and FF of group 3 and the elevated Cu levels in FF of oocytes of the highest quality from PCOS women warrant further analyses. Moreover, a decreased Zn concentration had been described before in PCOS, potentially related to insulin resistance and hyperandrogenemia [42].

Our data accord well with previous studies describing relatively low concentrations of Cu and Zn in FF as compared to serum [43]. Similarly, total average Se concentrations and GPX3 activity were reported to be lower in FF as compared to serum [44,45]. However, the average differences reported vary, and in the recent analysis of *n* = 103 couples from China, serum Se was about 50% higher in serum than in FF (108 ± 21 vs. 71 ± 15 µg/L) [44], whereas this difference was much more pronounced in our samples from Austrian women (serum; 67 µg/L vs. FF; 24 µg/L, Figure 3). This finding may relate to the relatively poor Se status in Austria and other areas of central Europe [46] and points to a Se-dependent gradient from serum to FF. In serum, it is known that the two major circulating selenoproteins, namely GPX3 and SELENOP, are reaching certain saturated expression levels upon sufficient Se intake that are even used to define an optimal Se status [34,47,48]. It can be assumed that there are similar mechanisms operating in FF in order to tightly control and limit Se status and selenoprotein levels, as both GPX3 and SELENOP were successfully detected in FF and correlated linearly to serum levels. However, the optimal concentrations in FF are unknown, as the samples analyzed were derived from European women with insufficient habitual intake, much in contrast to, e.g., subjects residing in North America where Se intake is higher due to better soil Se status [49,50].

To our surprise, the TE concentrations differed strongly between the FF from the same woman, even more intensively than among all the serum samples from the 40 women analyzed in this study. This result is in some agreement with a recent report on essential and non-essential TE in FF from the United States, where the differences were associated with demographic and clinical parameters, such as age, race, or BMI [51]. Unfortunately, data from matched serum samples of these U.S. American women are currently not available. Alternative influences on TE in FF may be exerted by follicle size and maturity [52]. This parameter is of likely little relevance to the results reported in our study, as follicle size was controlled for, and the follicles chosen displayed uniformly a minimal diameter of 15 mm; smaller follicles were not used for our analysis.

Our results indicate elevated TE concentrations in the groups of follicles with higher quality in comparison to FF without an oocyte, largely in agreement with several prior studies [45]. However, some reports also indicate adverse effects with increasing TE concentrations, e.g., for Cu in FF in relation to ongoing pregnancy [43] or for elevated Cu in PCOS in relation to steroidogenesis [53]. Again, these data support the notion of a tightly controlled and physiological relevant microenvironment for the developing oocyte, including not only the required supply with micronutrients but also protection from surplus and potentially toxic concentrations of TE.

In addition to the traditional scoring and selection of embryos using microscopy on predefined days, we also evaluated through time-lapse imaging the morphokinetic parameters of early embryo development. Several publications have proposed additional morphological evaluations to assess the timing of embryonic cell divisions that appear to be related to embryo viability [54,55,56,57,58]. Especially, the duration of the second cell cycle (cc2) and the synchrony of the second and third cell divisions (s2, s3) were described to be important indicators correlating with the chance of embryo implantation [10]. Here, we observed, as expected, that the morphologically defined oocyte group with the highest quality (group 5) was the one synchronizing the fastest. In addition, the cell cycle duration was considerably shorter. Comparable studies reported similar results, with shorter s2 and s3 in embryos forming blastocysts compared to those with poor quality [59]. On the other hand, an accelerated synchronization without changing morphokinetic dynamics was detected in patients suffering from endometriosis [32].

It is tempting to speculate that a specific time frame for synchronization favors a successful development process in early embryo morphokinetics. Group 4 displayed extended times for cell division, which led to distorted embryo development. A distortion of morphokinetic embryo behavior was also reported for women suffering from PCOS [60]. In our study, no such differences in morphokinetic development were observed between PCOS and the other group, which may be due to the small sample size. The TE determined in our study correlated well with certain morphokinetic variables, especially ECC2, s2, and s3, along with the inverse correlation of Se with ECC2 in embryos degenerated after fertilization. These findings point to an as yet unexplained relevance of TE for successful development and the high importance of an optimal TE status for successful fertilization and embryo development. A dysregulated Se status may hinder the accurate timing of cell division, thereby leading to alterations in optimal morphokinetic cleavage times. The slight but consistently lower Zn concentrations in serum and FF of women with PCOS may warrant attention and further investigation, as suboptimal Zn concentrations negatively affect several aspects of female fertility, including meiosis and fertilization competence of the egg [61]. Observational studies are in agreement with this notion, as Zn deficiency was associated with a longer time to achieve pregnancy in women actively planning to conceive [62]. Animal experiments have indicated some positive effects of Zn supplementation on fertility in a rat model of PCOS, but whether these preclinical results can be directly translated to human patients with PCOS remains to be evaluated [63].

Among the particular strengths of our study are the established and validated technologies used, the high quality of the biobank, and the TE quantifications that were conducted at a remote site from the biobank by scientists blinded to the clinical characteristics. Hereby, unbiased analysis and interpretation were enabled, and single FF with matched serum samples could be successfully studied without the need for analyzing pooled samples only. The findings indicate that not only do the TE concentrations vary strongly between FF from the same woman, but also the expression of physiological relevant protein biomarkers of TE status such as SELENOP and GPX3 differ. In view of their essential role in controlling redox milieu and oxidative stress, it is conceivable that Se deficiency correlates to poor oocyte quality. Among the limitations of our analyses are the small group sizes of serum samples analyzed. However, relatively suitable matching of the groups and of FF with serum was successfully achieved, and the number of FF analyzed was relatively high.

## 5. Conclusions

We conclude that there is a direct relationship between TE in serum and FF and that, in particular, the three biomarkers of Se status correlate positively in FF, highlighting their similar suitability for follicle-specific Se status assessment. The mechanisms connecting TE in serum and FF are poorly understood, in particular in view of the high variability between different FF obtained from the same woman at the same time. As the FF with the poorest quality showed a trend to the lowest TE concentrations, we conclude that TE deficiencies should be avoided during ART and oocyte development. It is likely that FF does not control their TE status by simple filtration of serum but rather by highly regulated and feedback-controlled mechanisms likely involving endocrine effects on uptake and maintenance of TE and the TE-dependent proteins and enzymes to ensure an optimal microenvironment for oocyte development.

## Figures and Tables

**Table 1:** Demographic and clinical characteristics of the women undergoing assisted reproduction.

	Subfertile		PCOS		
Parameter	Mean ± SD	*n*	Mean ± SD	*n*	*p*-Value *
Age (year)	35 ± 3	20	29 ± 4	20	<0.0001
BMI (kg/m^2^)	22 ± 3	20	23 ± 3	20	n.s.
Smoker/non-smoker	-	2/18	-	4/16	
Hormonal status					
FSH (mIU/mL)	6.4 ± 1.6	19	6.6 ± 2.0	19	n.s.
AMH (ng/mL)	3.5 ± 2.2	19	8.2 ± 4.6	17	0.0004
E2 (pg/mL)	38.0 ± 15.1	19	38.3 ± 15.5	19	n.s.
Newborn height and weight					
Newborn height (cm)	48 ± 4	7	50 ± 5	5	n.s.
Newborn weight (g)	2994 ± 863	7	3001 ± 809	5	n.s.
ICSI/IVF/missing data	19/1/0		15/3/2		

* unpaired *t*-test. n.s.; not significant.

**Table 2:** TE concentrations in FF of different quality from women with or without PCOS.

		Subfertile		PCOS		
Group		Mean ± SD	*n*	Mean ± SD	*n*	*p*-Value
1	Se (µg/L)	23 ± 10	17	19 ± 11	13	n.s.
	Cu (µg/L)	555 ± 191	17	518 ± 267	13	n.s.
	Zn (µg/L)	474 ± 118	17	402 ± 164	13	n.s.
	GPX3 (U/L)	96 ± 33	17	96 ± 49	13	n.s.
2	Se (µg/L)	31 ± 26	8	24 ± 11	10	n.s.
	Cu (µg/L)	683 ± 289	8	682 ± 381	10	n.s.
	Zn (µg/L)	569 ± 144	8	572 ± 231	10	n.s.
	GPX3 (U/L)	103 ± 25	8	102 ± 42	10	n.s.
3	Se (µg/L)	24 ± 14	10	22 ± 5	5	n.s.
	Cu (µg/L)	516 ± 185	10	479 ± 179	5	n.s.
	Zn (µg/L)	571 ± 111	10	419 ± 142	5	0.039
	GPX3 (U/L)	86 ± 35	10	75 ± 12	5	n.s.
4	Se (µg/L)	26 ± 11	16	24 ± 11	17	n.s.
	Cu (µg/L)	619 ± 176	16	652 ± 276	17	n.s.
	Zn (µg/L)	536 ± 170	16	467 ± 174	17	n.s.
	GPX3 (U/L)	111 ± 36	16	103 ± 40	17	n.s.
5	Se (µg/L)	22 ± 11	13	27 ± 12	15	n.s.
	Cu (µg/L)	507 ± 190	13	742 ± 318	15	0.028
	Zn (µg/L)	503 ± 167	13	591 ± 220	15	n.s.
	GPX3 (U/L)	97 ± 38	13	105 ± 38	15	n.s.
Δ (2–1)	Se (µg/L)	9 ± 17	8	7 ± 9	6	n.s.
	Cu (µg/L)	179 ± 283	8	236 ± 389	6	n.s.
	Zn (µg/L)	138 ± 120	8	270 ± 285	6	n.s.
	GPX3 (U/L)	21 ± 45	7	8 ± 25	5	n.s.
Δ (3–1)	Se (µg/L)	2 ± 11	8	3 ± 7	4	n.s.
	Cu (µg/L)	75 ± 189	8	28 ± 152	4	n.s.
	Zn (µg/L)	128 ± 167	8	31 ± 205	4	n.s.
	GPX3 (U/L)	0 ± 42	8	−22 ± 13	3	n.s.
Δ (4–1)	Se (µg/L)	2 ± 9	13	4 ± 9	13	n.s.
	Cu (µg/L)	46 ± 190	13	132 ± 223	13	n.s.
	Zn (µg/L)	83 ± 161	13	49 ± 143	13	n.s.
	GPX3 (U/L)	9 ± 39	12	3 ± 26	11	n.s.
Δ (5–1)	Se (µg/L)	1 ± 8	10	9 ± 12	11	n.s.
	Cu (µg/L)	27 ± 141	10	274 ± 277	11	0.02
	Zn (µg/L)	−28 ± 169	10	164 ± 215	11	0.036
	GPX3 (U/L)	3 ± 31	9	10 ± 42	9	n.s.

n.s.; not significant.

**Table 3:** Morphokinetic times in oocytes categorized into groups 4 (good quality) and 5 (best quality).

	Group 4		Group 5		
Parameter	Mean ± SD	*n*	Mean ± SD	*n*	*p*-Value
ECC2 (h)	13.6 ± 6.0	19	12.6 ± 1.5	19	n.s.
ECC3 (h)	22.7 ± 7.3	15	19.1 ± 3.5	15	n.s.
S2 (h)	5.2 ± 5.8	20	1.8 ± 2.6	20	0.023
S3 (h)	15.7 ± 9.1	15	8.7 ± 4.8	15	0.017

## Data Availability

The data presented in this study are available on request from the corresponding author. The data are not publicly available due to data safety reasons.

## Supplementary Materials

The following are available online at https://www.mdpi.com/article/10.3390/nu13114134/s1, Table S1: Correlation analysis between trace elements and GPx3 activity with morphokinetic parameters.
