# Supplementary material for: Pronounced Trace Element Variation in Follicular Fluids of Subfertile Women Undergoing Assisted Reproduction
Source: Nutrients. 2021 Nov 19;13(11):4134. doi: 10.3390/nu13114134 (PMC8619388; doi:10.3390/nu13114134)
Supplement: Supplementary file 1 [file nutrients-13-04134-s001.zip › nutrients-1431752-supplementary.pdf]

Table S1. Correlation matrices between trace elements and GPx3 activity with morphokinetic parameters.

**Copper**	ECC2_g4	ECC2_g5	ECC3_g4	ECC3_g5	S2_g4	S2_g5	S3_g4	S3_g5	Cu_ECC2_g4	Cu_ECC2_g5	Cu_ECC3_g4	Cu_ECC3_g5	Cu_S2_g4	Cu_S2_g5	Cu_S3_g4	Cu_S3_g5
ECC2_g4	1															
ECC2_g5	-0,35	1														
ECC3_g4	-0,031	0,346	1													
ECC3_g5	-0,215	-0,03	-0,16	1												
S2_g4	0,559	-0,049	0,194	-0,064	1											
S2_g5	0,018	0,143	0,232	-0,135	0,032	1										
S3_g4	-0,37	0,44	0,765	0,01	0,211	0,087	1									
S3_g5	-0,168	0,182	-0,064	0,549	-0,053	0,532	0,041	1								
Cu_ECC2_g4	-0,117	0,274	0,288	0,492	-0,047	-0,173	0,414	0,15	1							
Cu_ECC2_g5	0,207	0,272	0,355	0,055	0,343	-0,22	0,241	-0,264	0,501	1						
Cu_ECC3_g4	-0,238	0,449	0,214	0,306	-0,048	-0,164	0,436	-0,014	0,946	0,604	1					
Cu_ECC3_g5	0,222	-0,004	0,314	0,012	0,321	-0,209	0,17	-0,284	0,495	0,995	0,596	1				
Cu_S2_g4	-0,173	0,273	0,248	0,497	-0,063	-0,173	0,411	0,154	1	0,504	0,98	0,498	1			
Cu_S2_g5	0,19	0,26	0,141	0,073	0,31	-0,266	0,03	-0,235	0,333	0,995	0,375	0,985	0,337	1		
Cu_S3_g4	-0,215	0,45	0,262	0,3	-0,034	-0,165	0,443	-0,02	0,98	0,6	1	0,593	0,981	0,369	1	
Cu_S3_g5	0,222	-0,004	0,314	0,012	0,321	-0,209	0,17	-0,284	0,495	0,995	0,596	1	0,498	0,985	0,593	1

**Zinc**	ECC2_g4	ECC2_g5	ECC3_g4	ECC3_g5	S2_g4	S2_g5	S3_g4	S3_g5	Zn_ECC2_g4	Zn_ECC2_g5	Zn_ECC3_g4	Zn_ECC3_g5	Zn_S2_g4	Zn_S2_g5	Zn_S3_g4	Zn_S3_g5
ECC2_g4	1															
ECC2_g5	-0,35	1														
ECC3_g4	-0,031	0,346	1													
ECC3_g5	-0,215	-0,03	-0,16	1												
S2_g4	0,559	-0,049	0,194	-0,064	1											
S2_g5	0,018	0,143	0,232	-0,135	0,032	1										
S3_g4	-0,37	0,44	0,765	0,01	0,211	0,087	1									
S3_g5	-0,168	0,182	-0,064	0,549	-0,053	0,532	0,041	1								
Zn_ECC2_g4	-0,2	0,205	0,129	0,475	-0,105	-0,08	0,325	0,296	1							
Zn_ECC2_g5	0,027	0,402	-0,022	0,076	0,021	0,057	-0,031	0,125	0,194	1						
Zn_ECC3_g4	-0,149	0,434	0,116	0,467	0,097	0,026	0,391	0,281	0,924	0,581	1					
Zn_ECC3_g5	0,061	0,327	0,116	0,018	0,085	0,029	0,095	0,024	0,231	0,969	0,654	1				
Zn_S2_g4	-0,21	0,206	0,134	0,485	-0,104	-0,081	0,337	0,304	0,999	0,198	0,933	0,233	1			
Zn_S2_g5	-0,018	0,393	-0,017	0,093	0,027	0,05	-0,014	0,141	0,174	0,997	0,529	0,959	0,182	1		
Zn_S3_g4	-0,132	0,431	0,113	0,451	0,094	0,025	0,382	0,268	0,934	0,572	0,999	0,65	0,941	0,516	1	
Zn_S3_g5	0,061	0,327	0,116	0,018	0,085	0,029	0,095	0,024	0,231	0,969	0,654	1	0,233	0,959	0,65	1

**Selenium**	ECC2_g4	ECC2_g5	ECC3_g4	ECC3_g5	S2_g4	S2_g5	S3_g4	S3_g5	Se_ECC2_g4	Se_ECC2_g5	Se_ECC3_g4	Se_ECC3_g5	Se_S2_g4	Se_S2_g5	Se_S3_g4	Se_S3_g5
ECC2_g4	1															
ECC2_g5	-0,35	1														
ECC3_g4	-0,031	0,346	1													
ECC3_g5	-0,215	-0,03	-0,16	1												
S2_g4	0,559	-0,049	0,194	-0,064	1											
S2_g5	0,018	0,143	0,232	-0,135	0,032	1										
S3_g4	-0,37	0,44	0,765	0,01	0,211	0,087	1									
S3_g5	-0,168	0,182	-0,064	0,549	-0,053	0,532	0,041	1								
Se_ECC2_g4	-0,423	0,286	0,147	0,251	-0,315	-0,112	0,475	0,098	1							
Se_ECC2_g5	-0,2	0,39	0,262	-0,126	0,044	-0,158	0,322	-0,192	0,48	1						
Se_ECC3_g4	-0,427	0,327	0,113	0,11	-0,285	-0,124	0,478	-0,064	0,981	0,543	1					
Se_ECC3_g5	-0,191	0,126	0,13	-0,17	-0,049	-0,163	0,209	-0,208	0,431	0,994	0,492	1				
Se_S2_g4	-0,427	0,285	0,148	0,258	-0,314	-0,112	0,477	0,104	1	0,486	0,982	0,436	1			
Se_S2_g5	-0,269	0,371	0,367	-0,095	0,042	-0,075	0,445	-0,157	0,582	0,993	0,641	0,979	0,589	1		
Se_S3_g4	-0,42	0,328	0,111	0,102	-0,286	-0,125	0,476	-0,072	0,982	0,536	1	0,486	0,982	0,633	1	
Se_S3_g5	-0,191	0,126	0,13	-0,17	-0,049	-0,163	0,209	-0,208	0,431	0,994	0,492	1	0,436	0,979	0,486	1

**GPx3**	ECC2_g4	ECC2_g5	ECC3_g4	ECC3_g5	S2_g4	S2_g5	S3_g4	S3_g5	Gpx_ECC2_g4	Gpx_ECC2_g5	Gpx_ECC3_G4	Gpx_ECC3_g5	Gpx_S2_g4	Gpx_S2_g5	Gpx_S3_g4	Gpx_S3_g5
ECC2_g4	1															
ECC2_g5	-0,35	1														
ECC3_g4	-0,031	0,346	1													
ECC3_g5	-0,215	-0,03	-0,16	1												
S2_g4	0,559	-0,049	0,194	-0,064	1											
S2_g5	0,018	0,143	0,232	-0,135	0,032	1										
S3_g4	-0,37	0,44	0,765	0,01	0,211	0,087	1									
S3_g5	-0,168	0,182	-0,064	0,549	-0,053	0,532	0,041	1								
Gpx_ECC2_g4	-0,346	0,265	0,378	0,198	-0,055	-0,131	0,694	0,062	1							
Gpx_ECC2_g5	0,063	0,193	0,647	-0,162	0,215	-0,184	0,512	-0,373	0,489	1						
Gpx_ECC3_G4	-0,454	0,356	0,243	-0,028	0,02	-0,248	0,651	-0,12	0,955	0,53	1					
Gpx_ECC3_g5	0,065	0,075	0,48	-0,248	0,048	-0,1	0,309	-0,316	0,291	0,992	0,322	1				
Gpx_S2_g4	-0,349	0,265	0,379	0,201	-0,055	-0,13	0,696	0,065	1	0,487	0,955	0,289	1			
Gpx_S2_g5	-0,003	0,191	0,675	-0,14	0,218	-0,149	0,565	-0,357	0,526	1	0,547	0,992	0,526	1		
Gpx_S3_g4	-0,445	0,333	0,232	-0,029	0,048	-0,27	0,648	-0,108	0,955	0,529	1	0,307	0,955	0,542	1	
Gpx_S3_g5	0,065	0,075	0,48	-0,248	0,048	-0,1	0,309	-0,316	0,291	0,992	0,322	1	0,289	0,992	0,307	1

Color code: red indicates inverse, blue positive correlations; color intensity indicates strength of correlation.
